# Cardiac autonomic involvement in Huntington’s disease

**DOI:** 10.1007/s10072-024-07428-5

**Published:** 2024-03-04

**Authors:** Dilek İşcan, Yakup Çetinkaya

**Affiliations:** 1 Department of Neurology, Faculty of Medicine, Niğde Ömer Halisdemir University, 51240 Niğde, Turkey; 2 Department of Cardiology, Faculty of Medicine, Niğde Ömer Halisdemir University, 51240 Niğde, Turkey

**Keywords:** Huntington’s disease, Cardiac autonomic dysfunction, Heart rate variability

## Abstract

**Introduction:**

Huntington’s disease (HD) is known as a neurodegenerative disease with movement disorder and cognitive impairment; autonomic involvement is also becoming common in some recent studies. The aim of this study is to demonstrate the presence of cardiac autonomic involvement in HD patients.

**Method:**

Time and frequency domain parameters obtained from the 24-h Holter ECG(hECG) were compared between 20 HD patients and 20 healthy control subjects.

**Results:**

Fourteen HD patients had tachycardia, bradycardia, and extra beats. Interval between two heartbeats, normal-to-normal (NN), standard deviation of all normal-to-normal (SDNN), square root of the mean of the sum of the squares of the differences between consecutive N-N intervals in ms (rMSSD), and the ratio of the number of consecutive pairs of N-N intervals that differ by more than 50 ms to the total number of N-N intervals (pNN50) were all significantly higher in the patient group than in the control group during 24-h hECG monitoring. However, hECG monitoring showed that the patient group had significantly higher values of the frequency-domain metrics high frequency (HF) than the control group did (*P* = 0.003). Very low frequency (VLF) was lower in the patient group (*P* = 0.009). There was no difference in low frequency (LF) in both groups. In comparison to the control group, LF/HF was much reduced in the patient group (*P* = 0.001).

**Conclusion:**

Cardiac disfunction increases, and autonomic functions change in HD, but more comprehensive studies are needed to distinguish sympathetic and parasympathetic involvement.

## Introduction

Cytosine-adenine-guanine (CAG) trinucleotide repeat expansions in exon-1 of the Huntingtin gene (HTT) are the cause of Huntington’s disease (HD), an inherited neurological condition that runs in the autosomal dominant mode [1]. As a result of the meta-analysis, the incidence of HD was calculated to be 0.38/100,000 and the prevalence to be 2.71/100,000 [2]. With its influence on cellular proteostasis, axonal transport, transcription, translation, mitochondrial, and synaptic processes, the mutant HTT gene causes neuronal malfunction and death [3]. HD is marked by significant striatal disease [4].

With escalating physical, cognitive, and mental symptoms, HD often begins in middle life and has catastrophic repercussions on the patient and his or her family. Rapid**,** uncontrollable, twisting movements of the face, torso, and extremities are the most recognizable symptoms of HD. Dystonia, rigidity, and bradykinesia are other HD symptoms that may exist. In addition, HD patients may have a slow down in their psychomotor and executive abilities as well as a regression in their memory, emotional, and social-cognitive domains [5]. Autonomic involvement in HD is a relatively understudied topic. Although HD has primarily been linked to mobility disorder, cognitive dysfunction, and mental symptoms, some research has suggested that HD may also influence the autonomic nervous system [6, 7]. It has been hypothesized that HD autonomic dysfunction is caused by anatomical and functional abnormalities in the central autonomic network of the brain [8]. In fact, HD patients reported gastrointestinal, urinary, sexual, and cardiac problems more frequently than control participants did [9]. In relation to HD, reports of abnormal sympathetic and parasympathetic nervous system activity have been made [10]. The aim of this study was to show the presence of cardiac autonomic dysfunction and its relationship with disease duration in HD patients.

## Method

### Patient selection and study design

The patient group consisted of patients who had a family history of HD and who presented with HD symptoms to the neurology outpatient clinic at Nigde Omer Halisdemir Training and Research Hospital between December 10, 2021 and October 30, 2022. Nigde Omer Halisdemir University Faculty of Medicine Non-Interventional Clinical Research Ethics Committee approved the study’s protocol before it was carried out (Decision No: 2022-108).

Detailed physical examinations, Holter electrocardiography (hECG) monitorization, and genetic testing all revealed that they had HD. The control group, on the other hand, was made up of healthy individuals who underwent a hECG examination and were matched in terms of gender and age to the patients in the patient group. They were also free from any active neurological, cardiological, or autonomic complaints. The age range was not specified when choosing patients so order to assure randomization. The study’s inclusion criteria were a diagnosis of HD and completion of a hECG; its exclusion criteria were the absence of a genetic diagnosis of HD, the absence of medical records and a family history, the failure to complete a hECG, under the age of 18 years, the presence of conditions known to affect heart rate variability (HRV), and the use of medications like beta-blockers and calcium channel blockers that affect HRV. Demographic information, including age, gender, and place of birth, was recorded for both patients and controls. Patients’ family history, age at disease onset, and disease duration were all properly recorded.

### Cardiac assessment

#### Holter electrocardiography

All patients had a three-lead DMS9800 + 3 model hECG device implanted at 14.00 and removed at 14.00 the following day. During the 24-h hECG recording, both the sick and the healthy control participants were instructed to carry on with their regular activities. An expert cardiologist who was blinded to the patients and control individuals reexamined and reviewed the hECG records. The gathered information was examined using the Ambulatory ECG System application. The American College of Cardiology/American Heart Association (ACC/AHA) ECG and ambulatory ECG clinical compliance report was used to analyze the hECG results [11]. Data were gathered on the mean 24-h heart rate, HRV, arrhythmias, and conduction disturbances. The autonomic status was assessed in two domains, namely the time domain and the frequency domain, and HRV was measured using a hECG.

The R-R interval on an ECG refers to the space between two successive R waves. The R-R distance of QRS complexes coming from the sinus node is calculated as N-N (normal-to-normal). Calculations in the time domain are performed by computing changes in N-N intervals. The square root of the mean of the sum of the squares of the differences between consecutive N-N intervals in ms is rMSSD, and the ratio of the number of consecutive pairs of N-N intervals that differ by more than 50 ms to the total number of N-N intervals is pNN50 (%50). The standard deviation of all NN intervals is SDNN, the standard deviation of normal N-N intervals in 5-min segments over 24 h is SDNN index, and the power values correlate to frequency values stated in ms^2^. In the spectrum, frequencies below 0.04 Hz are referred to as very low frequency (VLF), and they represent the renin-angiotensin-aldosterone system, thermoregulation, and vasomotor tonus. Low frequency (LF) in the spectrum denotes sympathetic tonuses and is defined as 0.04–0.15 Hz. High frequency (HF) is also defined as 0.15–0.40 Hz in the spectrum, which denotes parasympathetic activation. A rise in the LF/HF number implies that sympathetic activity is predominating.

### Statistical analysis

The data were analyzed with SPSS 26.0 (Statistical Product and Service Solutions for Windows, Version 26.0, IBM Corp., Armonk, NY, USA, 2019) package program. Continuous variables were reported as mean ± standard deviation (X ± SD), median, interquartile range values, and categorical variables as number (*n*) and percentage (%). The Kolmogorov-Smirnov test was employed to identify whether the data was distributed normally. When parametric test assumptions were provided, an independent *t* test was used to compare independent group differences, and when parametric test assumptions were not provided, the Mann-Whitney *U* test was employed to compare independent group differences. *P* < 0.05 was regarded as the level of significance as the result of statistical tests.

## Results

The study comprised 20 HD patients—11 (55%) females and 9 (45%) males—as well as 20 healthy control subjects—11 (55%) females and 9 (45%) males. The mean ages of the patient and control groups were, respectively, 57.10 + 8.83 (range 42 to 70) and 53.33 + 6.58 (range 40 to 66) years (*P* = 0.188). The average illness lasted 10.35 ± 5.04 (range 4 to 20) years. At the time of the disease’s onset, the average patient was 46.75 ± 6.83 years old (range 36 to 57), with female patients being 45.54 years old and male patients being 48.22 years old.

The hECG of the patient group showed that four patients had ventricular extrasystole, three had sinusoidal tachycardia, three had supraventricular tachycardia, three had sinusoidal bradycardia, and one patient had both supraventricular and ventricular extra beats. On the other hand, no one in the control group had tachycardia or bradycardia, according to the hECG.

The mean heart rates (HR) in the patient and control groups were, respectively, 83.65 ± 16.82 and 73.75 ± 9.49 beats per minute (*P* = 0.027).

With the help of 24-h hECG monitoring, it was possible to compare the time-domain parameters SDNN, SDNN index, rMSSD, and pNN50 between the patient and control groups, as shown in Table 1. These comparisons showed that the patient group had significantly higher SDNN, rMSSD, and pNN50 values than the control group did. On the other hand, utilizing 24-h hECG monitoring to compare frequency-domain metrics, such as HF, LF, VLF, and LF/HF parameters between the patient and control groups, it was found that the patient group had considerably greater HF and lower VLF, LF/HF than the control group (*P* < 0.05). There was no significant relationship between disease duration and time domain and frequency domain parameters (*P* > 0.05). No significant difference was detected between holter parameters in the groups with and without arrhythmia (*P* > 0.05).

**Table 1:** Comparison of the time- and frequency-domain parameters between the patient and control groups

Variables	Patient group (*n*: 20) Median (IQR)	Control group (*n*: 20) Median (IQR)	*z*	*p* value
SDNN (ms)	215.00 (526.25)	131.50 (47.50)	− 2.665	0.008*
SDNN index (ms)	63.50 (56.50)	53.00 (15.75)	− 1.408	0.159
rMSSD (ms)	44.50 (41.00)	25.00 (14.25)	− 3.114	0.002*
pNN50	15.50 (20.75)	4.50 (8.75)	− 2.576	0.010*
Triangle	22.50 (12.50)	24.00 (6.50)	− 0.664	0.507
HF	434.55 (609.40)	155.20 (215.70)	− 3.003	0.003*
LF	391.90 (368.03)	561.30 (352.68)	− 1.569	0.117
LF/HF	0.94 (0.83)	3.29 (2.88)	− 4.436	0.001*
VLF	742.90 (657.65)	939.55 (388.68)	− 2.597	0.009*

*SDNN* standard deviation of all normal-to-normal intervals, *SDNN index* standard deviation of normal N-N intervals in 5-min segments over 24 h, *rMSSD* the square root of the mean of the sum of the squares of the differences between consecutive normal-to-normal intervals, *pNN50* ratio of the number of consecutive pairs of normal-to-normal intervals differing by more than 50 ms to the total number of normal-to-normal intervals, *HF* high frequency, *LF* low frequency, *VLF* very low frequency, *z* Mann-Whitney *U* test, *p* significance between groups

^*^Probability (*p*) < 0.05

As shown in Table 2, when the time and frequency domain parameters of sleep and wakefulness were evaluated in 11 patients with HD and 12 patients from the healthy control group, the HF value of the patient group during sleep was significantly higher than that of the control group (*P* = 0.042), the LF/HF value of the patient group was lower during sleep and wakefulness compared to the control group (*P* < 0.05), and the VLF value of the patient group was lower during wakefulness (*P* = 0.027). In the patient group, rMSSD, pNN50, HF, LF, LF, and VLF were significantly lower during wakefulness than during sleep; in the healthy control group, rMSSD, LF, and LF/HF were significantly lower during wakefulness than during sleep.

**Table 2:** Comparison of time-and-frequency-domain parameters between the patient and control groups according to sleep-wake up time

Variables	Patient group (*n*: 11) Median (IQR)	Control group (*n*: 12) Median (IQR)	*p*^2^
SDNN (ms)			
W*akefulness*	155.63 (124.35)	96.50 (19.00)	0.157
*Sleep*	105.56 (185.11)	106.06 (46.25)	0.460
*p*^1^	1.000	0.814	
SDNN index (ms)			
*Wakefulness*	65.26 (105.53)	51.00 (26.90)	0.157
*Sleep*	63.03 (115.30)	56.50 (26.10)	0.295
*p*^1^	0.286	0.06	
rMSSD (ms)			
*Wakefulness*	34.78 (33.05)	25.75 (18.95)	0.196
*Sleep*	37.81 (32.56)	29.00 (16.75)	0.085
*p*^1^	0.010*	0.008*	
pNN50			
*Wakefulness*	10.85 (25.32)	5.00 (10.90)	0.157
*Sleep*	12.24 (31.24)	7.74 (11.30)	0.096
*p*^1^	0.013**	0.099	
HF			
*Wakefulness*	293.61 (619.13)	157.50 (255.38)	0.140
*Sleep*	327.55 (537.35)	150.00 (179.75)	0.042*
*p*^1^	0.05*	0.410	
LF			
*Wakefulness*	365.80 (396.29)	421.50 (452.75)	0.498
*Sleep*	525.50 (466.52)	650.00 (492.25)	0.712
*p*^1^	0.010*	0.023*	
LF/HF			
*Wakefulness*	1.22 (1.87)	2.97 (1.72)	0.010*
*Sleep*	1.27 (1.60)	3.10 (3.74)	0.003*
*p*^1^	0.657	0.041*	
VLF			
*Wakefulness*	546.51 (580.59)	1175.00 (1041.75)	0.027*
*Sleep*	941.43 (1058.83)	1164.00 (667.50)	0.268
*p*^1^	0.05*	0.530	

*SDNN* standard deviation of all normal-to-normal intervals, *SDNN index* standard deviation of normal N-N intervals in 5-min segments over 24 h, *rMSSD* the square root of the mean of the sum of the squares of the differences between consecutive normal-to-normal intervals, *pNN50* ratio of the number of consecutive pairs of normal-to-normal intervals differing by more than 50 ms to the total number of normal-to-normal intervals, *HF* high frequency, *LF* low frequency, *VLF* very low frequency, *p1* Wilcoxon test, *p2* Mann-Whitney *U* test

^*^Probability (*p*) < 0.05

## Discussion

To demonstrate the presence of cardiac autonomic involvement in HD, a 24-h hECG was inserted in 20 patients diagnosed with HD and 20 healthy control groups. HRV is an effective noninvasive method for detecting cardiac autonomic dysfunction. In this context, time-domain parameters, such as SDNN, SDNN index, rMSSD, and pNN50, and frequency-domain parameters, such as HF, LF, VLF, and LF/HF, were compared between HD patients and healthy controls. SDNN, rMSSD, and p50 time domain parameters were significantly higher in the patient group. HF, one of the frequency domain parameters, was higher in the patient group. LF/HF was lower in the patient group than in the control group; this result was partially attributed to elevated HF. VLF was lower in the patient group. There was no difference in the time domain during sleep and interval between patient and healthy control groups, but HF was high in the patient group during sleep and VLF was low during wakefulness.

In the study by Cutler et al. with mouse, young Huntington’s mouse had abnormally high HR late in the day and early at night, while middle-aged mice had low HR. When active, during daylight hours, HR was also elevated in young and middle-aged mutant mouse [12]. In a study by Schultz et al. comparing HD patients and controls at rest, heart rate was higher in the patient group than in the control group [13]. This has been associated with decreased functional connectivity in the central autonomic system as a result of the degenerative process. It has been hypothesized that there is a decrease in parasympathetic activity in HD [14]. In our study, HR was high in the patient group in accordance with these studies.

Vagal activity is reflected by time-domain parameters. Reduced rMSSD indicates decreased vagal activity and vagal nuclei degeneration in the central nervous system [15, 16]. On the other hand, heart rate variability can be determined using periodic heart rate oscillations at various frequencies, i.e., the five frequency bands between 0 Hz and 0.5 Hz, LF, MF, HF, ULF, and VLF (VLF). LF represents sympathetic system activity, while HF represents parasympathetic system activity. Consequently, the LF/HF ratio represents relative sympathetic dominance [16, 17]. It has been hypothesized that neurodegenerative processes affecting both cortical and subcortical structures and associated with HD may selectively affect the autonomic control network [18]. In contrast to the study of Andrich et al., parasympathetic tone was found to be increased in our study.

Changes in autonomic function may also result from peripheral causes. HD is associated with peripheral tissue abnormalities. Many of these abnormal alterations appear to result directly from the expression of mutant preying in peripheral tissues, although not all are caused by brain dysfunction. By the results of this study, Marotta et al. found, in a study involving 30 HD patients, that time-domain parameters, such as HRV, mean heart rate, SDNN, and RMSSD, were significantly higher, and frequency-domain parameters, such as HF, were significantly lower in awake stage in the patient group compared to the control group. In addition, mean heart rate, a time-domain parameter, and frequency-domain parameters, i.e., LF and LF/HF, were significantly higher in the non-rapid eye movement (non-REM) sleep stage in the patient group than in the control group, indicating that the ongoing sympathetic tone was prevalent during non-REM sleep stages in HD. Except for SDNN, which was substantially lower in the patient group, indicating a decreased parasympathetic modulation and impaired circadian rhythm, they did not observe any differences between the patient and control groups during the REM sleep stage [19]. The hypothalamus, which controls the circadian rhythm, has been demonstrated to be involved in HD [20]. SDNN, rMSSD, pNN50, and HF were significantly higher in the patient group compared to the control group, and were lower in the LF/HF and VLF patient group. LF is increased in HD patients during sleep.

Terroba-Cambi et al. did not find a statistically significant difference between the groups in HRV in a study involving HD patients categorized into groups with single and recurrent falls. Nonetheless, after the 6-month follow-up, the rMSSD while at rest and LF/HF while standing were significantly associated with recurrent fall risk in the subsequent 6 months [21]. Considering that the patients who fell had more advanced stages of the disease, we can comment that Terroba-Cambi did not find a relationship between early stage and late stage. Autonomic changes have been shown to start 25 years before the symptoms of the disease [13]. Although we did not compare patients who fell and did not fall in our study, we did not find a relationship between the duration of the disease and HRV.

Kle et al., in their study, stated that the most important parameter predicting mortality within the frequency domain parameters is VLF [22]. When our data was evaluated, VLF was significantly lower in HD patients. Unlike other studies, we thought that cardiac mortality in HD may be higher than in the normal population. However, we could not find the causes of cardiac mortality due to the small number of patients and technical reasons.

In the study of Kobal et al., sympathetic hyperfunction was found in asymptomatic gene carriers and mildly disabled HD patients, but autonomic hypofunction was found in advanced HD patients [7]. In our study, mean HR, SDNN, rMSSD, pNN50, HF, and LF/HF were significantly higher in the patient group. Maybe, we detected it this way at an early stage because our patients were generally mobilizable.

In the study of Bär and et al., ECG recordings were made for 5 min each while resting and moving in 12 HD patients and the control group. There was no significant difference for RMSSD, LF, and HF parameters when patients at rest and controls were compared, while mean heart rate and LF/HF were significantly higher in patients [23]. While there was no change in any parameter in the control group when standing up, mean heart rate and LF/HF increased significantly, and rMSSD decreased significantly in the patient group. Unlike this study, in our study, there was no significant change in LF/HF during the daytime, i.e., when patients were active, and rMSSD increased. Since we recorded 24 h, the recording may have been affected by many variables.

This study has some limitations. First of all, we did not perform sympathetic skin responses, deep breathing (valsalva maneuver), or drug administration. Although it is a rare disease, the small number of patients may be considered a limitation. More comprehensive and multicenter studies will provide much more important information on this subject.

## Conclusion

Arrhythmia was observed in more than half of the patients with Huntington’s disease, and significant differences were detected in Holter parameters compared to healthy controls. Unlike many previous studies, our study is valuable because it included 24-h recordings and patients continued their daily activities. We wanted to emphasize that Huntington’s patients should have cardiology follow-ups as well as Neurology and Psychiatry follow-ups.
